# Eating Behaviour Predicts Weight Loss Six Months after Bariatric Surgery: A Longitudinal Study

**DOI:** 10.3390/nu10111616

**Published:** 2018-11-02

**Authors:** Kavitha Subramaniam, Wah-Yun Low, Peng-Choong Lau, Kin-Fah Chin, Karuthan Chinna, Nik Ritza Kosai, Mustafa Mohammed Taher, Reynu Rajan

**Affiliations:** 1 Medical Education, Research and Development Unit, Faculty of Medicine, University of Malaya, Kuala Lumpur 50603, Malaysia; eskei13@yahoo.co.uk; 2 Department of Physical and Mathematical Science, Faculty of Science, Tunku Abdul Rahman University, Kampar, Perak 31900, Malaysia; 3 Faculty of Medicine Dean’s Office, University of Malaya, Kuala Lumpur 50603, Malaysia; 4 Department of Surgery, Faculty of Medicine, University of Malaya, Kuala Lumpur 50603, Malaysia; laupc@ummc.edu.my (P.-C.L.); mdskfc@gmail.com (K.-F.C.); 5 Department of Social and Preventive Medicine, Faculty of Medicine, University of Malaya, Kuala Lumpur 50603, Malaysia; karuthan@gmail.com; 6 Minimally Invasive, Upper Gastrointestinal and Bariatric Surgery unit, Department of surgery, Faculty of Medicine, Universiti Kebangsaan Malaysia Medical Centre (UKMMC), Universiti Kebangsaan Malaysia, Kuala Lumpur 56000, Malaysia; nikkosai@ppukm.ukm.edu.my (N.R.K.); mutafa@ppukm.ukm.edu.my (M.M.T.); dr.reynu@gmail.com (R.R.)

**Keywords:** obesity, weight loss, bariatric surgery, eating behaviour, psychology

## Abstract

Bariatric surgery is currently the most durable weight loss solution for patients with morbid obesity. The extent of weight loss achieved, however, is subject to variation due to various factors, including patients’ behaviour. In this study, we aimed to identify pre- and post-surgical predictors of weight loss following bariatric surgery. This prospective study included 57 participants who went through bariatric surgery (laparoscopic Roux-en-Y gastric bypass: *n* = 30; laparoscopic sleeve gastrectomy: *n* = 23; one anastomosis gastric bypass-mini gastric bypass: *n* = 4) in two tertiary referral hospitals. Consenting participants were assessed prior to surgery (T_0_), and three months (T_1_) and six months (T_2_) after surgery. The assessment included interview and anthropometric measurements. The interview was done with the aid of instruments, including the Hospital Anxiety and Depression Scale (HADS) for anxiety and depression screening and the Dutch Eating Behaviour Questionnaire (DEBQ) for eating behaviour assessment. Baseline comorbidity status was obtained from medical records. A Generalised Estimating Equation (GEE) was developed to determine predictors of weight loss. Participants in the study were mostly women (*n* = 37, 65%) with a mean age of 39.4 (SD = 10.01) years. The mean excess BMI loss (EBMIL) and total weight loss (TWL) at the sixth month was 63.31% and 23.83%, respectively. Anxiety, depression, and external eating scores reduced over time. Advancing age, high BMI, and higher scores for emotional and external eating emerged as significant negative predictors for TWL%. It can be concluded that the patients experienced substantial weight loss after surgery. Continuous monitoring of psychological well-being and eating behaviour are essential for optimal weight loss.

## 1. Introduction

The global prevalence of obesity has increased greatly over the past four decades [1], with a dramatic rise in the rates of morbid and severe obesity [2]. Currently, bariatric surgery is the most durable weight loss solution for patients with morbid obesity. It enables an average loss of up to 60–70% of excess weight, and long-term maintenance of the loss [3,4]. Despite the impressive average weight loss values, there is individual variation in the rate of weight loss; around 10–30% of the patients have suboptimal weight loss or weight-regain [5,6]. This has motivated research on predictors of successful weight loss, to identify behavioural traits that are important for better weight loss outcome and development of post-surgical interventions.

The existing studies have identified a myriad of pre- and post-surgical predictors of weight loss, which remain inconclusive, until now. The impact of gender is a factor that has been previously addressed, and the findings vary from a favourable outcome among women [7] and suboptimal loss among men [8], to absence variation in the achievement of excess weight loss (EWL) [9] and maintenance of weight loss [6] across gender groups. A study that compared the analyses on surgical outcomes performed using cross-sectional, as well as a longitudinal analytical technique, reported that cross-sectional comparison showed higher EWL values among women, whereas longitudinal regression with weight as outcome showed that women lost less weight, as compared to men, across time [10]. This finding cautions that interpretation of the role of gender should be made by taking into account possibilities, such as women having lower initial weight and, consequently, having achieved higher EWL values. Younger participants were found to have better weight loss outcomes [9,11]. Participants of Black Ethnic group were found to have lower weight loss compared to Caucasians at the 6th and 12th month after surgery [7].

Obesity related co-morbidities were reported to affect weight loss. Patients with diabetes mellitus [12,13] were found to have achieved lower weight loss averages, as compared to those without diabetes and, among those with diabetes, patients who received insulin therapies had poorer outcome [12]. Elevated level of triglycerides and HbA1C were also reported to affect weight loss outcome [9]. The HbA1C level, or the glycaemic control, was found to be a stronger predictor of weight loss than being diagnosed for diabetes [9]. Mixed findings have been reported when psychiatric morbidity is concerned. The combination of the presence of two or more psychiatric disorders, no eating disorders, and having adverse childhood experiences, was found to be associated with low average weight loss [14]. Contradicting evidence was reported in other studies, in which higher depression scores were associated with lesser risk of weight regain [6], and pre-surgical history of mood disorders were associated with better weight loss outcome [15]. In a review study on the impact of psychopathology on weight loss, the authors concluded that post-operative—and not the pre-operative—psychopathology affected weight loss, as the former affects one’s flexibility and ability to adapt to post-surgical behavioural changes [16]. This explains the reported favourable association between psychopathology and weight loss. An earlier review study showed that presence of obesity-related psychopathology, such as mild depression and low self-esteem, did not impose a detrimental effect on weight loss, but serious psychiatric morbidities, such as major depression and personality disorders, did, as these conditions impose difficulties in adapting to necessary post-surgical behavioural changes [17]. Recent evidence further supported that negative personality traits were associated with lower weight loss outcome [11].

Adherence to the dietary recommendations provided was shown to be an important predictor of successful weight loss outcome [18]. Presence of disordered eating behaviours, such as grazing [18] and bingeing [19], were associated poorer weight loss outcomes. Interestingly, among those who had low adherence to recommended dietary regiments, those who grazed not more than once per day had a weight loss success rate of 68.3%, as compared to 27.6% among those who grazed more than once [18]. These findings show the interconnectedness of the behavioural traits, that should be noted while monitoring patients. Emotional and uncontrolled eating traits were associated with lower weight loss achievements [20]. In addition to that, behaviours such as frequent clinical visits [18] and regular exercise [21] were associated with better weight loss.

The abovementioned studies provided valuable input on factors that affected weight loss after surgery. The variations in type of surgery, duration of follow-up, time of assessment, and assessment methods, have contributed greatly to the inconsistencies in the findings. Studies of cross-sectional nature do not account for the change in behaviour; regression models for weight loss outcome based on pre-surgical psychological or behavioural parameters do not leave the readers with much clue on the impact of post-surgical factors. Similarly, models based on behaviours at post-surgical timelines do not provide much information on necessary behavioural changes. With regards to those findings, the current study was designed to identify the trends in weight loss and predictors of total weight loss (TWL), six months after surgery, with behavioural predictors assessed over time. The duration of six months was selected to determine if the behavioural variables, especially modifiable variables, have an impact on weight loss at the acute weight loss phase, when a dramatic weight loss is expected. Preventive measures could be drafted to handle such modifiable predictors, in order to optimise weight loss during the period at which rapid weight loss is expected. In addition to that, patients have frequent contacts with healthcare providers during the first six months after surgery and, thus, could be easily monitored.

## 2. Materials and Methods

### 2.1. Participants

Participants of the study were patients with obesity who underwent bariatric surgery for weight loss in two tertiary referral hospitals in Kuala Lumpur, Malaysia, between December 2011 to December 2016 in one hospital, and January 2016 to January 2017 in the other. Consenting participants were assessed thrice, before surgery (T_0_), and three months (T_1_) and six months (T_2_) after surgery. Follow-up interviews were mostly conducted during clinical appointments. During the clinical appointment, patients met the surgeons who evaluated their recovery after surgery, dietitians who advised them on dietary plans, and sports medicine specialists who advised them on essential exercises. The follow-up interviews for this study, which evaluated their psychological improvements, were not part of the patient care regime, and were carried out for research purposes only. The participants who had no appointments at a suitable duration were either met elsewhere or interviewed over the telephone. Those who refused both the options were classified as not available for the particular interview. Participants who were not available for any follow-up were excluded.

In total, 80 participants were recruited upon obtaining written consent and, of them, 57 (71.25%) completed at least one follow-up interview, and were included in the study. Forty-five (56.25%) of the 80 participants turned up for the second interview, and 43 (53.75%) turned up for the third interview. A total of 36 (63% of those included in the study) participants had completed all three follow-ups. The surgical interventions given were laparoscopic Roux-en-Y gastric bypass 30 (53%), laparoscopic sleeve gastrectomy 23 (40%), and laparoscopic one anastomosis gastric bypass–mini gastric bypass 4 (7%).

### 2.2. Measures

The participants of the study were interviewed, and subjected to height and weight measurements during the assessments. Medical records were reviewed to obtain information on co-morbidity and type of surgery. The Hospital Anxiety and Depression Scale [22] was used to screen for anxiety and depression. The recommended cut-off score for the anxiety and depression subscales were 7–8 and above, for possible anxiety and depression; 10–11 and above, for probable anxiety and depression; and a score of 14–15 and above, for severe anxiety and depression. This instrument has shown good validity and reliability for the Malaysian population [23].

Eating behaviour was assessed with the Dutch Eating Behaviour Questionnaire (DEBQ) [24]. This questionnaire contains 33 items that assess three types of eating behaviours: (i) emotional eating—eating in response to emotional cues [25], (ii) external eating—eating in response to external food-related cues [26], and (iii) dietary restraint (restrained eating)—dietary control via cognitive cues with the intention to lose weight [27]. Higher scores for each subscale indicated stronger behavioural traits. The DEBQ instrument had satisfactory structural validity and reliability for our Malaysian samples [28].

Basic sociodemographic information collected include gender, age, ethnicity, marital status, occupation, housing, and mode of transport used. Two items, housing and mode of transport, were included as additional socioeconomic indicators, as a person’s socioeconomic status is influenced by the income of the nucleus family, and one’s occupation, alone, may not be adequate to depict it. Family history of obesity was also recorded. Information on the presence of obesity-related co-morbidities, including diabetes, hypertension, dyslipidemia, fatty liver disease, sleep apnoea, and conditions that caused difficulty in walking, was obtained from patients’ medical records.

Height and weight measurements were taken during follow-ups, where height was measured at the nearest 0.1 cm, and weight to the nearest 0.1 kg, using the standard scales in the hospitals. Body mass index (BMI) was estimated as (weight in kg)/(height in m)^2^. Total weight lost (TWL)% was estimated as ((preoperative weight − postoperative weight)/preoperative weight) × 100%. Excess BMI loss, EBMIL, was estimated as follows: ((pre-treatment BMI − current BMI)/(pre-treatment BMI − 25)) × 100%. Ideal weight was the weight that had a BMI of 25.

The EWL and EBMIL are the standard weight matrices reported in almost all bariatric literature. The EBMIL was calculated across timelines to compare the findings with existing literature. TWL was used to study the weight loss pattern and predictors of weight loss instead of the EWL/EBMIL, since the BMI of 25, which is used to estimate EBMIL and EWL, is not the optimal BMI cut-off for an Asian population. That is because, for Asians, a higher risk of chronic diseases was reported at a lower BMI than for Caucasians [29,30]. The World Health Organisation (WHO) Expert Consultation did not suggest a different BMI cut-off for the entire diverse Asian population. The panel of experts, however, suggested BMI 23 kg/m^2^ to be considered as high risk, and 27.5 kg/m^2^ as very high risk for public health intervention [31]. In line with these, the value of BMI 25 in the EBMIL formula was not amended for the current study. A measure that is non-dependent on any cut-off criteria will be a better option to identify predictors of weight loss in this case. The TWL was least associated with pre-surgical weight [32], an important predictor of post-surgical weight [10,32]. Using TWL, the impact of other modifiable variables could be identified, which will be helpful in patient selection and management, to optimise weight loss after surgery.

### 2.3. Ethics Clearance

Ethical approval was obtained from the ethics committee of both hospitals (MEC Ref No 732.19; JEP-2016-276). Written informed consent was obtained from all participants during the first interview.

### 2.4. Statistical Analysis

Friedman’s test was used to compare the change of distribution across timelines, and Wilcoxon’s sign rank test with Bonferroni’s adjustment was used to conduct post hoc comparisons. Kendall’s W was used for effect size estimates. Effect size measured by Kendall’s W is considered small if the value is 0.1, moderate if 0.3, and strong if 0.5 and above. Mann–Whitney *U* test was used to compare the cross-sectional difference between groups. Spearman’s correlation co-efficient value was used to determine the strength of association between the variables. Factors associated with TWL were studied using a generalised estimating equation (GEE). The GEE was used instead of the linear mixed model, which was used in previous studies, and deemed suitable to study the impact of weight loss [10]. This is because the quasi-likelihood estimation employed by the GEE, unlike the restricted maximum likelihood estimation used in the mixed model, is not stringent on normality assumption [33,34]. The distribution of weight loss and other psychological variables, in this study, were found to deviate from normality. The GEE is a better option for such datasets [34]. Pre-surgical as well as time-varying (from pre to post) variables were included in the model. The pre-surgical factors included were age, gender, ethnicity, marital status, family history of obesity, initial BMI, and baseline co-morbidities. The time-varying factors tested were longitudinal BMI, eating behaviour, anxiety, and depression, across the study duration. Variables that were statistically significant (*p* < 0.05) and improved overall fit of the model were retained.

## 3. Results

### 3.1. Demographic and Health Information

The participants in this study (*n* = 57) formed 71.0% of the total surgical patients recruited. The characteristics of the participants and dropout group were compared. It was found that the two groups were similar in terms of demography and weight distribution (Table 1). The study group was representative of the cohort of patients who went through surgery. The participants in the study were mostly Malay and women, with an average age of 39.40 years (SD = 10.01) and average initial BMI of 45.52 kg/m^2^ (SD = 9.94) (Table 1). The Non-Malay group that formed a substantial minority of 25% (*n* = 15), consisted of Indians (*n* = 10, 17.5%), Chinese (*n* = 2, 3.5%), Caucasian (*n* = 1, 1.75%), and a Pakistani (*n* = 1, 1.75%). The Caucasian and Pakistani participants had resided in Malaysia for a long time (over three decades for the Caucasian participant; the Pakistani participant was born and brought up in Malaysia) and had Malaysian citizenship. Most participants were living in their own houses and had their own transportation. Most participants (*n* = 35, 61%) were employed as professionals, associate professionals, or in managerial and executive positions (Table 1).

### 3.2. Weight Loss

**Figure 1:**
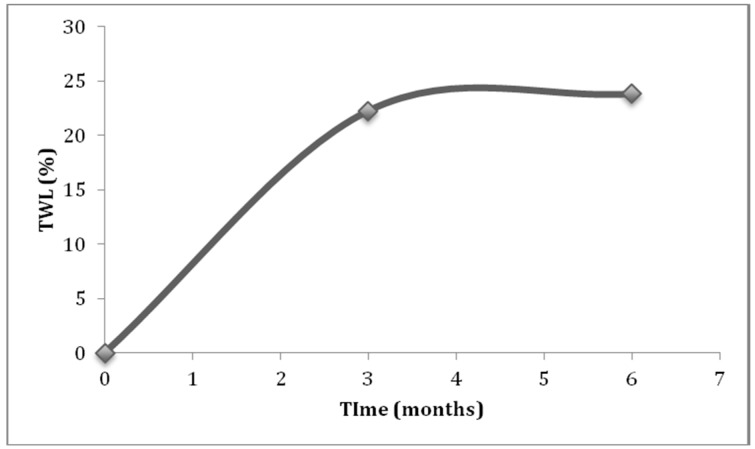
Total weight loss (TWL) experienced across the study duration.

The mean weight and BMI loss experienced in the first three months after surgery were 20.52 kg (SD = 7.95) and 7.57 kg/m^2^ (SD = 2.62), whereas between the fourth to sixth month, they were 9.88 kg (SD = 10.61) and 3.59 kg/m^2^ (SD = 3.61). Post hoc comparisons showed that weight and BMI differed significantly across all timelines, and had a large effect size (Table 2). The average EBMIL and TWL achieved at the third month were 40.53% and 22.28%, and at the sixth month, they were 63.33% and 23.83%. The most impactful weight loss occurred during the third month. The participants of the study, who were given different surgical interventions, did not differ in terms of initial BMI (*Z* = 0.470, *p* = 0.638) and weight (*Z* = −0.157, *p* = 0.876). Patients who had laparoscopic Roux-en-Y gastric bypass (LRYGB) or one anastomosis gastric bypass–mini gastric bypass (MGB) performed on them had higher EBMIL and TWL at the third and the sixth month, as compared to those who had LSG performed. A significant difference (*p* < 0.05) was only observed in the value of TWL between LRYGB and LSG patients at the sixth month (T_2_) (Table 3). The MGB group was not included in the analyses, due to it having a small sample size. Change of TWL across time formed a steep line from 0 to 3 months, that almost flattened from the third to sixth month (Figure 1).

### 3.3. Psychological Factors and Eating Behaviour

Changes in psychological variables across time are shown in Table 4. Anxiety scores decreased over time, with a significant reduction between baseline and the sixth month. The prevalence rate of anxiety was 21% at T_0_, and dropped to 7% at T_1_ and 3.5% at T_2_. Depression scores reduced significantly between baseline and the third month, as well as between baseline and the sixth month. The prevalence rate of depression at baseline was 7%, and was reduced to 5.3% at T_1_ and 1.8% at T_2_. Changes in anxiety and depression had a moderate effect size (Table 4).

External eating was the only eating behaviour that changed over time, with a significant reduction between baseline and the third month, as well as between baseline and the sixth month. The change had a moderate effect size (Table 4). The emotional eating scores across time were positively correlated. A similar observation was obtained for the external eating scores. There was also a positive correlation between the emotional and external eating scores. Restrained eating at T_2_ correlated with emotional eating across all timelines (Table 5). The emotional and external eating scores at T_2_ were correlated with anxiety and depression across almost all timelines (Table 6). The external and emotional eating scores, due to the correlation between them, could not be regressed together. Two separate GEE models were developed to explain the impact of the variables on TWL.

### 3.4. Predictors of Weight Loss Following Surgery

The GEE models show that the average monthly TWL experienced was 5.7% (6.12*t* − 0.42*t^2^*) (Table 6) and 5.3% (5.68*t* − 0.38*t*^2^) (Table 7). The squared term for time had a negative value, indicating a quadratic equation with an increasing trend (inverted *U* pattern). In the first model, increased age, higher BMI, and higher emotional eating scores were found to be associated with lower TWL percentages (Table 7). The second model showed that increased age, higher BMI, and higher external eating scores were associated with lower TWL percentages (Table 8). All other predictors (except eating behaviours) in both the models were similar, and had slope values that were almost similar.

Depression, which was found to be a significant predictor of TWL after controlling for age, BMI and time, became insignificant upon inclusion of emotional and external eating in the equations. Eating behaviours were found to be stronger psychological predictors than depression. Anxiety, initial weight, initial BMI, comorbidities, family history of obesity, and other sociodemographic factors, were not significantly associated with change in TWL%.

## 4. Discussion

This study examined the weight loss and predictors of weight loss at six months post-surgery. The mean EBMIL experienced in the six months was 63.33%, which was around the values reported for the same duration in previous studies, 56.4% [7] and 65% [13]. The mean TWL at the sixth month, 23.83%, was also around the value reported for six months in another study, 25.7% [7]. The weight loss achieved by the participants in this study is comparable to what is reported elsewhere.

Initial weight is a predictor for weight loss that has been replicated in many studies. Models with EWL/EBMIL as the outcome showed that higher initial weight (or BMI) were negative predictors of weight loss outcome [7,9,13,35], and the impact was seen at the sixth month post-surgery [7,13]. It has been shown that initial weight was the most important predictor that explained over 93% of the variation in weight [36]. In this study, initial BMI was not a significant predictor of TWL. A previous study that used TWL as the outcome also reported an absence of association between initial weight and TWL [37]. The negative slope obtained for the continuous BMI variable, however, still leads to a similar conclusion of an inverse association with higher weight and percentage of weight loss. The negative relationship could be due to a lower level of physical activities among heavier patients [36]. In addition, large absolute values of weight loss experienced by these patients, when converted to percentage of total body weight lost, could be projected as a smaller value, as compared to a person with lower body weight.

The inverse association between increased age and weight loss, found in this study, is in coherence with previous findings [9,11]. Lower rates of co-morbidities among younger patients [16], decreased physically activity among older adults [38], and slower recovery with increasing age, could contribute to the age effect. The slope value of *β* = −0.10 and *β* = −0.12 for age shows that TWL is reduced by 0.1 or 0.12 units, with the increase in age by one unit. A patient who is 60 years old is expected to experience between 6% and 7.2% less weight loss due to age, whereas a 40-year-old person experienced between 4% and 4.8% less weight loss. A difference of 2% and 2.4%, for a 20-year age gap, is not a formidable amount. Age should not be a reason for older patients being denied this surgical option, considering the many improvements in co-morbidity and metabolism that followed [39].

The observed positive correlations between emotional eating scores across time indicate that higher scores at pre-surgical time-line were indicative of higher score post-surgically. A similar trend was seen for external eating. These findings show that the eating behaviour traits do not disappear during the early stages after surgery and affect weight loss during the rapid weight loss period. The findings could be related to a previous study which showed that emotional eating was associated with suboptimal weight loss (failure to achieve EWL > 50%) two years post-surgery [20]. A study with a 10-year follow-up showed that those who had lost <10% EWL, or regained weight, had higher uncontrolled eating and hunger scores [40]. The uncontrolled eating or disinhibition was described as eating opportunistically, or in relation to external food-related cues [41], and overlaps, to an extent, with the concept of external eating. The previous findings could, thus, be related to the current finding that eating in response to external stimuli, be it emotional or food-related cues, has an adverse effect on weight loss outcome post-surgery. The observed correlation between restrained eating at T_2_, and emotional eating at all time lines, could potentially refer to the effort that the patients with emotional eating take to control their diet, probably due to the unsatisfactory weight loss percentages achieved.

A review showed that emotional eating, in addition to having a direct negative impact on weight loss, was also found to be the underlying reason for various disordered eating behaviours; such as uncontrolled eating, grazing, and binge eating [42]. Those behaviours were found to be induced by a variety of emotional triggers [42]. A recent finding showed that the emotional and external eating behaviours were associated with depression [43]. These lines of evidence, that showed the existence of a complex relationship between eating behaviour and psychopathology, are further supported by our findings, which show that emotional and external eating, six months after surgery, were correlated with anxiety and depression scores.

The clinical implication of the current finding is that eating behaviours affect weight loss during the acute weight loss period. It is a stronger negative predictor than depression scores at the early stage. However, it is of note that psychopathology was low among the participants, due to the patient selection process for surgery, where patients with severe psychiatric morbidity were excluded. Patients with tendencies to eat in response to external cues, be it emotion or food-related stimuli, should be identified and subjected to counselling and behavioural therapy, and given continuous support to curb the behaviours which are detrimental to weight loss. Acceptance-based behavioural treatments, which have been shown to be effective in improving negative eating behaviours and causing better weight loss or maintenance [44], could be adapted for such surgery candidates. It is of utmost importance to note that the eating behaviour traits were self-reported measures made using screening instruments and, thus, should not be used as a patient selection tool but, rather, an additional way of guiding the patients towards better weight loss outcome.

There are some limitations to the study. Firstly, the limited sample size has an inhibitive effect on the power to identify predictors with smaller impacts. The follow-up duration of six months was selected to study the impact of behavioural variables at an early stage. A longer follow-up duration is essential to observe the impact of behavioural factors on weight loss. The psychological factors and eating behaviour variables were self-reported by patients and, thus, the risks of under- or overreporting are undeniable. Future studies, with larger sample sizes, longer follow-up durations, and psychological and eating behaviour assessments by experts, are warranted. Despite these limitations, the current finding shows that the impact of psychobehavioural factors, at the early stage after surgery, is of importance for clinical management of patients and, thus, warrants attention.

## 5. Conclusions

The findings show that patients experienced significant weight loss and improvements in psychological factors after surgery. Eating behaviours were important predictors of weight loss six months post-surgery. Continuous assessment of eating behaviour during pre- and post-surgical follow-up is essential to achieve good weight loss outcomes.

## Figures and Tables

**Table 1:** Characteristics of surgical patients who were included and excluded in this study.

Factor	Included in the Study (*N* = 57) *n* (%)	Excluded from the Study (*N* = 23) *n* (%)	Test Statistics (χ^2^, *p*)
Gender			1.993, 0.123
Female	37 (64.9)	11 (47.8)	
Male	20 (27.0)	12 (52.2)	
Ethnicity			0.000, 0.592
Malay	43 (75.0)	17 (74.0)	
Non-Malays	14 (25.0)	6 (26.0)	
Age (years)			−0.039. 0.969 ^c^
Mean ± SD	39.40 ± 10.01	39.30 ± 10.98	
Median ± IQR ^a^	38.00 ± 15.00	39.00 ± 12.00	
Educational Qualification			1.248, 0.195
School ^b^	17 (30)	10 (43.5)	
College/University	39 (70)	13 (56.5)	
Housing Situation			0.748, 0.387
Own House	47 (82.5)	17 (74.0)	
Rented House/Quarters	10 (17.5)	6 (25.0)	
Mode of Transport			2.519, 0.112
Own Transport	52 (91.0)	18 (78.3)	
Public Transport/Others	5 (9.0)	5 (21.7)	
Occupation			NIL ^d^
Professional/Associate Professional	20 (35)	7 (30.4)	
Management and Executive	7 (12)	1 (4.3)	
Clerical Support	8 (14)	3 (13.0)	
Armed Forces	4 (7)	1 (4.3)	
Business Owners	5(9)	2 (9.0)	
Students	3 (5.4)	4 (17.0)	
Retired/At Home/Unemployed	10 (17.5)	5 (22.0)	
Initial BMI			1.060, 0.299 ^c^
Mean ± SD	45.52 ± 18.26	49.88 ± 18.29	
Median ± IQR ^b^	43.35 ± 12.33	45.52 ± 9.95	
Obesity Classification			
Class I	10 (17.50)	5 (22.00)	
Class II	6 (10.53)	3 (13.00)	0.105, 0.368 ^e^
Class III	41 (71.90)	15 (65.00)	

^a^ interquartile range; ^b^ inclusive primary school, secondary school, and religious school, where one participant belonged to the latter category; ^c^ independent sample *t*-test value and corresponding *p*-value is reported; ^d^ no comparison was made, due to the small sample size in each category; ^e^ chi-square value is obtained by categorising obesity Class I and II as one group (not-morbid obesity) and Class III (morbid obesity) as one group

**Table 2:** Weight changes across study duration.

Measure	Descriptive	Baseline (T_0_) (*n* = 57)	Third Month (T_1_) (*n* = 45)	Sixth Month (T_2_) (*n* = 43)	χ^2^	*p*	W
Weight (kg)	Mean, SD	122.33, 35.11	101.80, 31.74	91.93, 25.41	68.814	<0.001	0.930
	Median, IQR	114.0, 30.3	96.73, 22.70	87.00, 18.50			
BMI (kg/m^2^)	Mean, SD	45.48, 10.58	37.75, 9.86	34.11, 8.07	68.814	<0.001	0.930
	Median, IQR	42.453, 12.60	35.70, 8.52	32.39, 7.32			
TWL%	Mean, SD		22.28, 7.82	23.83, 7.40	NA		
	Median, IQR		22.45, 8.88	23.68, 7.71			
EWL%	Mean, SD		40.55, 18.33	63.33, 33.18	NA		
	Median, IQR		37.81, 23.11	57.83, 30.57			
EBMIL%	Mean, SD		40.55, 18.33	63.33, 33.18	NA		
	Median, IQR		37.81, 23.11	57.83, 30.57			

SD—standard deviation; IQR—interquartile range; TWL—total weight loss; NA—not applicable; EWL—excess weight loss; EBMIL—excess BMI loss; Friedman’s chi-square, *p*-value and the effect size, Kendall’s W are reported.

**Table 3:** Weight changes across study duration (by surgery type).

Surgery (mean, SD) (median, IQR)	EBMIL		TWL	
	T_1_ (*n* = 45)	T_2_ (*n* = 43)	T_1_(*n* = 45)	T_2_ (*n* = 43)
LRYGB (*n* = 30) (number followed-up)	41.89, 17.59	67.01, 36.91	17.07, 6.02	25.14, 6.98
	42.58, 21.05	58.13, 26.54	17.71, 6.84	24.97, 8.2
	*n* = 24	*n* = 28	*n* = 24	*n* = 28
LSG (*n* = 23)	38.87, 20.84	53.96, 28.26	15.74, 4.76	19.28, 6.33
	33.50, 23.44	45.97, 48.55	16.20, 8.60	19.84, 9.79
	*n* = 18	*n* = 11	*n* = 18	*n* = 11
MGB (*n* = 4)	42.76, 7.72	69.98, 13.55	18.29, 4.12	29.40, 7.52
	46.14, NA	72.66, 25.13	16.15, NA	27.99, 13.98
	*n* = 3	*n* = 4	*n* = 3	*n* = 4
*Z*, *p*^a^	−0.966, 0.334	−1.373, 0.170	−0.872, 0.383	−2.269, 0.023

LRYGB—laparoscopic Roux-en-Y gastric bypass; LSG—laparoscopic sleeve gastrectomy; MGB—one anastomosis gastric bypass–mini gastric bypass; SD—standard deviation; IQR—interquartile range; TWL—total weight loss; NA—not applicable; EWL—excess weight loss; EBMIL—excess BMI loss; ^a^ Mann–Whitney’s *U* test was conducted to compare the difference in EBMIL and TWL at the third and sixth month between surgical groups. The MGB group was not included, due to small sample size.

**Table 4:** Changes in psychological factors following surgery.

Measure Mean (SD)	T_0_	T_1_	T_2_	Test Statistics (χ*^2^*, *p*)	Effect Size (W)
Anxiety	4.88 (3.23)	3.43 (2.94)	2.40 (2.47)	32.53 **^,b^	0.465
Depression	3.72 (2.89)	2.02 (2.59)	1.60 (2.13)	28.82 **^,a,b^	0.412
Emotional eating	2.06 (0.94)	1.64 (0.80)	1.81 (0.81)	4.12 ^ns^	0.082
External eating	2.86 (0.68)	2.25 (0.79)	2.38 (0.64)	19.29 **^,a,b^	0.386
Restraint	2.65 (0.73)	2.93 (0.86)	2.75 (0.80)	3.49 ^ns^	0.070

** *p* < 0.01, ns—not significant, a—significant difference between T_0_ and T_1_, b—significant difference between T_0_ and T_2._

**Table 5:** Correlation between the eating behaviour traits across time.

	Emotional T_0_	Emotional T_1_	Emotional T_2_	External T_0_	External T_1_	External T_2_	Restrained T_0_	Restrained T_1_	Restrained T_2_
**Emotional T_0_**	1.00	0.452 **	0.787 **	0.484 **	0.302	0.623 **	−0.045	0.264	0.425 **
**Emotional T_1_**		1.00	0.808 **	0.312	0.763 **	0.709 **	0.277	0.239	0.489 *
**Emotional T_2_**			1.00	0.452 **	0.653 **	0.774 **	0.190	0.291	0.489 **
**External T_0_**				1.00	0.389 *	0.667 *	0.031	0.356 *	0.348
**External T_1_**					1.00	0.781 *	0.080	0.146	0.389
**External T_2_**						1.00	0.117	0.298	0.241
**Restrained T_0_**							1.00	0.304	0.381 *
**Restrained T_1_**								1.00	0.450
**Restrained T_2_**									1.00

* *p* < 0.05, ** *p* < 0.01.

**Table 6:** Correlation between anxiety, depression, and eating behaviour.

	Emotional T_0_	Emotional T_1_	Emotional T_2_	External T_0_	External T_1_	External T_2_	Restrained T_0_	Restrained T_1_	Restrained T_2_
Anxiety T_0_	0.078	0.343 *	0.484 **	0.180	0.104	0.325 *	0.230	0.357 *	0.430 **
Anxiety T_1_	0.210	0.382 *	0.473 **	0.009	0.232	0.347 *	0.500 **	0.254	0.390 *
Anxiety T_2_	0.345 *	0.575 *	0.569 **	0.291	0.414 *	0.573 **	0.157	0.457 *	0.216
Depression T_0_	0.102	0.169	0.377 **	0.234	0.092	0.341 *	0.309 *	0.189	0.387 *
Depression T_1_	−0.067	0.112	0.225	0.134	0.097	0.207	0.211	−0.071	0.234
Depression T_2_	0.355 *	0.3361	0.559 **	0.165	0.193	0.359 *	0.198	−0.017	0.277

Emotional—emotional eating, External—external eating, Restrained—restrained eating, Anxiety—anxiety score, Depression—depression score. T_0_—before surgery, T_1_—three months after surgery, T_2_—six months after surgery. * *p* < 0.05, ** *p* < 0.01.

**Table 7:** Predictors of total weight loss (TWL) following bariatric surgery.

Factor	Regression Coefficient, *β*	95% Confidence Interval of *β*	df
Intercept	12.67 **	(7.34, 18.00)	1
Time (month)	6.19 **	(5.15, 7.23)	1
Time^2^	−0.42 **	(−0.60, −2.34)	1
Age	−0.10 **	(−0.19, −0.02)	1
Emotional Eating	−0.32 **	(−0.57, −0.06)	1
BMI	−0.18 **	(−0.25, −0.10)	1

df—degrees of freedom. ** *p* < 0.01.

**Table 8:** Predictors of total weight loss (TWL) following bariatric surgery.

Parameter	Regression Coefficient, β	95% Confidence Interval	df
(Intercept)	19.196 **	(11.67, 26.73)	1
Time	5.688 **	(4.66, 6.73)	1
Time^2^	−0.376 **	(−0.55, −0.20)	1
Age	−0.122 **	(−0.21, −0.03)	1
BMI	−0.291 **	(−0.42, −0.16)	1
External	−0.344 *	(−0.62, −0.07)	1

df—degrees of freedom. * *p* < 0.05, ** *p* < 0.01.

## References

[1] Cesare M.D., Bentham J., Stevens G.A., Zhou B., Danaei G., Lu Y., Bixby H., Cowan M.J., Riley L.M., Hajifathalian K. (2016). Trends in adult body-mass index in 200 countries from 1975 to 2014: A pooled analysis of 1698 population-based measurement studies with 19.2 million participants. Lancet.

[2] Sturm R., Hattori A. (2013). Morbid obesity rates continue to rise rapidly in the United States. Int. J. Obes..

[3] Carter P.L. (2015). The evolution of bariatric surgery. Am. J. Surg..

[4] Madura J.A., Dibaise J.K. (2012). Quick fix or long-term cure? Pros and cons of bariatric surgery. F 1000 Med. Rep..

[5] Karlsson J., Taft C., Rydén A., Sjöström L., Sullivan M. (2007). Ten-year trends in health-related quality of life after surgical and conventional treatment for severe obesity: The SOS intervention study. Int. J. Obes..

[6] Odom J., Zalesin K.C., Washington T.L., Miller W.W., Hakmeh B., Zaremba D.L., Altattan M., Balasubramaniam M., Gibbs D.S., Krause K.R. (2010). Behavioral predictors of weight regain after bariatric surgery. Obes. Surg..

[7] Wise E., Hocking K.M., Kavic S.M. (2016). Predictors of excess weight loss after laparoscopic Roux-en-Y gastric bypass: Data from an artifical neural network. Surg. Endosc..

[8] Melton G.B., Steele K.E., Schweitzer M.A., Lidor A.O., Magnusin T.H. (2008). Suboptimal weight loss after gastric bypass surgery: Correlation of demographics, comorbidities, and insurance status with outcomes. J. Gastrointest. Surg..

[9] Ortega E., Morı R., Flores L., Moizem V., Rios M., Lacy A.M., Vidal J. (2012). Predictive factors of excess body weight loss 1 year after laparoscopic bariatric surgery. Surg. Endosc..

[10] Dallal R.M., Quebbemann B.B., Hunt L.H., Braitman L.E. (2009). Analysis of weight loss after bariatric surgery using mixed-effects linear modeling. Obes Surg..

[11] Piaggi P., Lippi C., Fierabracci P., Maffei M., Calderone A., Mauri M., Anselmino M., Cassano G.B., Vitti P., Pinchera A. (2010). Artificial neural networks in the outcome prediction of adjustable gastric banding in obese women. PLoS ONE..

[12] Campos G.M., Rabl C., Mulligan K., Posselt A., Rogers S.J., Westphalen A.C., Lin F., Vittinghoff E. (2008). Factors associated with weight loss after gastric bypass. Arch Surg..

[13] Still C.D., Wood G.C., Chu X., Manney C., Strodel W., Petrick A., Gabrielsen J., Mirshahi T., Argyropoulos G., Seiler J. (2014). Clinical factors associated with weight loss outcomes after Roux-en-Y gastric bypass surgery. Obesity..

[14] Kinzl J.F., Schrattenecker M., Traweger C., Mattesich M., Fiala M., Biebl W. (2006). Psychosocial predictors of weight loss after bariatric surgery. Obes. Surg..

[15] Sockalingam S., Hawa R., Wnuk S., Santiago V., Kowgier M., Jackson T., Okrainec A., Cassin S. (2017). Psychosocial predictors of quality of life and weight loss two years after bariatric surgery: Results from the Toronto Bari-PSYCH study. Gen. Hosp. Psychiatry.

[16] Wimmelmann C.L., Dela F., Mortensen E.L. (2014). Psychological predictors of weight loss after bariatric surgery: A review of the recent research. Obes. Res. Clin. Pract..

[17] Herpertz S., Keilmann R., Wolf A.M., Hebebrand J., Senf W. (2004). Do psychosocial variables predict weight loss or mental health after obesity surgery? A systematic review. Obes. Res..

[18] Robinson A.H., Adler S., Stevens H.B., Darcy A.M., Morton J.M., Safer D.L. (2014). What variables are associated with successful weight loss outcomes for bariatric surgery after 1 year?. Surg. Obes. Relat. Dis..

[19] Luiz L.B., Brito D.S., Debon M., Brandalise N., Azevodo J.T., Monbach K.D., Herberie S., Mottin C.C. (2016). Variation of beinge eating one year after Roux-en-Y gastric bypass and its relationship with excess weight loss. PLoS ONE.

[20] Novelli I.R., Fonseca L.G., Lopes D.G., Dutra E.S., Baiocchi de Carvalho K.M. (2018). Emotional eating behavior hinders body weight loss in women after roux-en-Y gastric bypass surgery. Nutrition.

[21] Egberts K., Brown W.A., Brennan L., O’Brien P.E. (2012). Does Exercise improve weight loss after bariatric surgery? A systematic review. Obes. Surg..

[22] Zigmond A.S., Snaith R.P. (1983). The Hospital Anxiety and Depression Scale. Acta. Psychiatr. Scand..

[23] Fatt Q.K., Atiya A.S., Heng N.G.C., Beng C.C. (2007). Validation of the hospital anxiety and depression scale and the psychological disorder among premature ejaculation subjects. Int. J. Impot. Res..

[24] Van Strien T., Frijters J.E.R., Bergers G.P., Defares P.B. (1986). The Dutch Eating Behaviour Questionnaire (DEBQ) for assessment of restrained, emotional and external eating behaviour. Int. J. Eat Disord..

[25] Bruch H. (1997). Obesity in childhood and personality development. Obes. Res..

[26] Schacter S., Goldman R., Gordon A. (1968). Effects of fear, food deprivation, and obesity on eating. J. Pers. Soc. Psychol..

[27] Herman C., Polivy J. (1980). Obesity.

[28] Subramaniam K., Low W.Y., Chinna K., Chin K., Krishnaswamy S. (2017). Psychometric properties of the Malay version of the Dutch Eating Behaviour Questionnaire (DEBQ) in a Sample of Malaysian Adults Attending a Health Care Facility. Malaysian J. Med. Sci..

[29] Chiu M., Austin P.C., Manuel D.G., Shah B.R., Tu J.V. (2011). Deriving ethnic-specific BMI cutoff points for assessing diabetes risk. Diabetes Care..

[30] He W., Li Q., Yang M., Jiao J., Ma X., Zhou Y., Song A., Heymsfield S.B., Zhang S., Zhu S. (2015). Lower BMI cutoffs to define overweight and obesity in China. Obesity..

[31] World Health Organization (WHO) Expert Consultations (2004). Appropriate body-mass index for Asian populations and its implication for policy and intervention strategies. Lancet..

[32] Hatoum I.J., Kaplan L. (2013). Advantage of percent weight loss as a method for reporting weight loss after Roux-en-Y gastric bypass. Obesity..

[33] Hedeker D., Gibbons R.D. (2006). Longitudinal Data Analysis.

[34] Naseri P., Majd H.A., Kariman N., Sourtiji A. (2016). Comparison of generalised estimating equation (GEE), mixed effects models (MEM) and repeated measure ANOVA in analysis of menorrhagia data. J. Paramed. Sci..

[35] Sillén L., Andersson E., Andersson E. (2017). Patient factors predicting weight loss after Roux-en-Y gastric bypass. J. Obes..

[36] Sczepaniak J.P., Owens M.L., Garner W., Dako F., Masukawa K., Wilson S.E. (2012). A simpler method for predicting weight loss in the first year after Roux-en-Y gastric bypass. J. Obes..

[37] Lai C., Aceto P., Petrucci I., Castelnuovo G., Callari C., Giustacchini P., Sollazzi L., Mingrone G., Bellantone R., Raffaelli M. (2016). The influence of preoperative psychological factors on weight loss after bariatric surgery: A preliminary report. J. Health Psychol..

[38] Hallal P.C., Andersen L.B., Bull F.C., Guthold R., Haskell W., Ekelund U. (2012). Global physical activity levels: Surveillance progress, pitfalls, and prospects. Lancet..

[39] Sjostrom L. (2013). Review of the key results from the Swedish Obese Subjects (SOS) trial—A prospective controlled intervention study of bariatric surgery. J Intern Med..

[40] Konttinen H., Peltonen M., Sjöström L., Carlsson L., Karlsson J. (2015). Psychological aspects of eating behavior as predictors of 10-y weight changes after surgical and conventional treatment of severe obesity: Results from the Swedish. Am. J. Clin. Nutr..

[41] Bryant E.J., King N.A., Blundell J.E. (2008). Disinhibition: Its effects on appetite and weight regulation. Obes. Rev..

[42] Chesler B.E. (2012). Emotional eating: A virtually untreated risk factor for outcome following bariatric surgery. Sci. World J..

[43] Sevinçer G.M., Konuk N., İpekçioğlu D., Crosby R.D., Cao L., Coskun H., Mitchell J.E. (2016). Association between depression and eating behaviors among bariatric surgery candidates in a Turkish sample. Eat Weight Disord..

[44] Forman E.M., Butryn M.L., Juarascio A.S., Bradley L.E., Lowe M.R., Herbert J.D., Shaw J.A. (2013). The Mind Your Health Project: A randomized controlled trial of an innovative behavioral treatment for obesity. Obesity.

